# Unveiling Cytotoxic Bioactive Compounds from *Erica carnea* L.: Extraction Optimization, Kinetic Modeling, and Biological Activity Assessment

**DOI:** 10.3390/plants14111679

**Published:** 2025-05-30

**Authors:** Violeta Jevtovic, Khulood Fahad Saud Alabbosh, Reem Ali Alyami, Maha Raghyan Alshammari, Badriah Alshammari, Pavle Mašković, Jelena Mašković, Jelena Nikolić, Milan Mitić

**Affiliations:** 1 Chemistry Department, College of Science, University of Ha’il, Ha’il 81451, Saudi Arabia; v.jevtovic@uoh.edu.sa (V.J.); reem-alyami1@hotmail.com (R.A.A.); maharaghyan@gmail.com (M.R.A.); badriah.alshammari@gmail.com (B.A.); 2 Biology Department, College of Science, University of Ha’il, Ha’il 81451, Saudi Arabia; k.alabosh@uoh.edu.sa; 3 Department of Chemical Engineering, Faculty of Agronomy, University of Kragujevac, Cara Dušana 34, 32102 Čačak, Serbia; pavlem@kg.ac.rs (P.M.); jelenav@kg.ac.rs (J.M.); 4 Department of Chemistry, Faculty of Science and Mathematics, University of Niš, 18000 Niš, Serbia

**Keywords:** cytotoxic activity, antioxidant activity, antimicrobial activity, thermodynamic parameters, unsteady-state diffusion model, Ponomarev model, natural source

## Abstract

The effects of temperature, time, and solvent concentration on the yield of bioactive compounds (BCs) with cytotoxic activity against three cell lines (Hep2c, RD, and L2OB) from *Erica carnea* L., as well as their influence on kinetic and thermodynamic parameters (enthalpy, entropy, and free energy), were investigated. The extract obtained at 30% ethanol, 50 °C, and 80 min showed the highest cytotoxic activity with IC_50_ values of 14.29, 13.93, and 22.23 µg/mL, respectively. The kinetics of BC extraction with cytotoxic activity was better described by the unsteady-state diffusion model compared to the Ponomarev model. The activation energy obtained was in the range of *Ea* = (4.92–26.57) kJ/mol. The thermodynamic parameters under transition theory at 50 °C were ∆S* = (−220.22)–(−285.15) J/Kmol, ∆H* = (2.24–23.88) kJ/mol, and ∆G* = (94.34–96.30) kJ/mol, indicating that the extraction of BCs with cytotoxic activity against the three cell lines is an irreversible and endothermic process. In addition to cytotoxic activity, the extracts demonstrated strong antioxidant activity (e.g., DPPH IC_50_ = 16.55 µg/mL) and antibacterial activity against multiple bacterial strains. Five antioxidant assays were applied, along with tests against eight bacterial strains for antibacterial activity. These findings suggest that *Erica carnea* L. is a promising natural source of multifunctional bioactive compounds with therapeutic potential.

## 1. Introduction

Medicinal plants have always been a valuable source of therapeutic agents and drug leads, including anticancer drugs. Naturally derived drugs are those containing pharmacologically active ingredients derived from biological or mineral sources, intended for use in the diagnosis, cure, mitigation, and prevention of diseases [1].

Phytochemicals from plant extracts represent an important source of natural products that have demonstrated excellent cytotoxic activities. However, plants from different origins exhibit diverse chemical compositions and bioactivities. Therefore, the discovery of plant-based anticancer agents from various parts of the world remains a challenging task [2].

*Erica carnea* L., or spring heath, is a perennial evergreen shrub belonging to the Ericaceae botanical family and the *Erica genus*. This plant can grow up to 50 cm in height, with leaves in the form of thick, short, and narrow needles. The flowers are grouped into clusters of blossoms, usually longer on one side, while the fruits are capsules containing seeds [3]. The plant primarily grows in Central and Southern Europe and South Africa, typically in mountainous regions and deciduous, coniferous, or mixed forests. In Serbia, it is found on the Tara Mountain [4].

Plant species of this family exhibit a wide range of biological activities, including antioxidant, antidiabetic, anti-inflammatory, antibacterial, and analgesic properties. Species from this botanical family have also been used in medicine for the treatment of urinary tract infections [5]. However, only a few studies have reported the beneficial properties of this plant [6,7]. To the best of our knowledge, there are no published data on the extraction process of phytochemicals from *Erica carnea* L.

Like other medicinal plants, *Erica carnea* L. can be directly used for its bioactive constituents. However, it is generally more effective to utilize extracts containing these bioactive compounds, as they often exhibit higher potency than the original plant material. Extraction plays a crucial role in making these compounds suitable for medicinal use [8,9].

The type and polarity of the extraction solvent are critical parameters that influence both the biological activity of the extract and the extraction yield. The choice of solvent must be tailored to the polarity and structure of the compounds present in the matrix being extracted. Among the solvents commonly used, ethanol has proven to be highly efficient for extracting phenolic compounds from various matrices. It is highly effective and serves as a safer alternative to toxic solvents [10,11].

In addition to the solvent, other factors that may influence the quality and yield of the extract include the extraction method, temperature, and extraction time [12,13]. Higher temperatures and longer extraction times can enhance mass transfer and decrease viscosity, thereby improving extraction efficiency; however, they may also cause degradation of the target compounds [14,15].

This study aimed to optimize the extraction of bioactive compounds from *Erica carnea* L. by examining how ethanol concentration, extraction time, and temperature affect extract quality and cytotoxic activity against three tumor cell lines (Hep2c, RD, and L2OB). Kinetic extraction models were also studied based on experimental data to understand the mechanisms governing the extraction process.

We also analyzed the extraction process thermodynamically to determine whether it is spontaneous, endothermic, and reversible, helping identify ways to improve efficiency. Furthermore, antioxidant and antibacterial activities of the extracts were evaluated to validate the biological activity of the extracted compounds.

## 2. Results and Discussion

### 2.1. Optimization of the Extraction of BCs from E. carnea L.

The independent variables studied were ethanol concentration (x1), extraction temperature (x2), and extraction time (x3). The dependent variables analyzed were the cytotoxic activity of the extract against Hep2c (y1), RD (y2), and L2OB (y3) cell lines. The results obtained using the 2^3^ experimental design are presented in Table 1.

ANOVA is used to determine the regression coefficients. The regression coefficients are presented in Table 2.

Each parameter was evaluated for statistical significance, with *p* > 0.05 indicating insignificance and *p* < 0.05 indicating significance. The coefficients of determination (*R*^2^), adjusted *R*^2^ (*R*^2^*adj*), and coefficient of variation (CV) were used as indicators of model quality. The ANOVA results are presented in Table 3.

#### 2.1.1. Hep2c Cell Lines

The experimentally obtained results for cytotoxic activity against Hep2c cell lines are presented in Table 2. The yield of cytotoxic activity ranged from 0.442 to 0.683. The highest yield was observed at 30% ethanol concentration, 50 °C, and 80 min. Conversely, the lowest yield was recorded at 70% ethanol concentration, 30 °C, and 20 min.

The effects of the parameters on cytotoxic activity against Hep2c cell lines are summarized in Table 2, while their significance, as determined using experimental design, is presented in Table 3.

Solvent concentration, extraction temperature, and extraction time had a statistically significant effect (*p* < 0.05, Table 4) on the concentration of BCs with cytotoxic activity against Hep2c. Table 3 results show a negative effect of ethanol concentration, while extraction time has a notably positive effect.

Among the factors studied, extraction time was the most significant variable, contributing 67.67%, followed by ethanol concentration (9.54%) and extraction temperature (5.85%). Regarding two-way interactions, it is worth noting how the combination of ethanol concentration and extraction time (x1x3), as well as the combination of extraction time and extraction temperature (x2x3), affected cytotoxic activity against Hep2c cell lines.

The three-way interaction (x1x2x3) also showed a significant influence (*p* < 0.05) on the yield of cytotoxic activity against Hep2c cell lines.

The predicted first-order polynomial model for cytotoxic activity against Hep2c cell lines is presented below. Factors and interactions that were assessed to be statistically insignificant at a significance level of 0.05 were omitted:

(1) $$y_{1}=0.5535-0.0142x_{1}+0.00847x_{2}+0.1007x_{3}-0.0075x_{1}x_{3}+0.0080x_{2}x_{3}+0.0072x_{1}x_{2}x_{3}$$

The predicted yield values for the corresponding cytotoxic activity against Hep2c cell lines are presented in Table 2. Based on the results shown in Table 2, it can be concluded that the predicted and experimentally obtained values were quite similar, indicating that the optimization was successful.

#### 2.1.2. RD Cell Lines

The yield values ranged from 0.421 to 0.716. Similarly, the highest yield was achieved at 30% ethanol concentration, 50 °C, and 80 min. For cytotoxic activity against RD cell lines, as shown in Table 4, ethanol concentration, extraction temperature, and extraction time were all significant (*p* < 0.05), contributing 30.83%, 7.16%, and 48.27%, respectively. Extraction time was particularly significant in achieving higher cytotoxic activity against RD cell lines. The interaction between solvent concentration and extraction time contributed 7.85%.

Table 3 shows that higher temperature and longer extraction time increased cytotoxic activity, while higher ethanol concentration decreased it. The negative influence of the linear term of ethanol concentration on cytotoxic activity against RD cell lines suggests a linear decrease in cytotoxic activity with increasing ethanol concentration.

The predicted first-order polynomial model for cytotoxic activity against RD cell lines (excluding factors and interactions assessed as statistically insignificant at a significance level of 0.05) is as follows:

(2) $$y_{2}=0.5806-0.0534x_{1}+0.0124x_{2}+0.0836x_{3}+0.00136x_{1}x_{3}$$

The predicted yield values for cytotoxic activity against RD cell lines are presented in Table 2. The similarity between the predicted and experimentally obtained values indicates that the optimization was successful.

#### 2.1.3. L2OB Cell Lines

The yield values ranged from 0.181 to 0.603. Similarly, the highest yield was achieved at 30% ethanol concentration, 50 °C, and 80 min. The effect of extraction parameters was examined to achieve maximum recovery of cytotoxic activity against L2OB cell lines (Table 3). The contributing factors were ranked as follows: extraction time (52.42%) > ethanol concentration (31.28%) > extraction temperature (5.54%) > interaction between ethanol concentration and extraction time (4.60%) > three-way interaction (2.67%).

The predicted first-order polynomial model for cytotoxic activity against L2OB cell lines (excluding factors and interactions assessed as statistically insignificant at a significance level of 0.05) is as follows:

(3) $$y_{3}=0.3792-0.0762x_{1}+0.0135x_{2}+0.1277x_{3}+0.0112x_{1}x_{3}+0.0065x_{1}x_{2}x_{3}$$

The predicted yield values for cytotoxic activity against L2OB cell lines are presented in Table 2. The similarity between the predicted and experimentally obtained values indicates that the optimization was successful.

#### 2.1.4. Fitting the Models

It is essential that the developed regression models (Equations (1)–(3)) provide an adequate approximation of real systems. In this study, a numerical method was used for validation. The numerical method employs the coefficient of determination (R^2^) and adjusted R^2^ (R^2^adj). Additionally, the coefficient of variation (CV) was calculated to assess model adequacy (Table 3).

The coefficient of determination (*R*^2^) represents the proportion of variation in the response attributed to the model rather than random error. For a well-fitted model, *R*^2^ should not be less than 80% [9]. When *R*^2^ approaches unity, it indicates a good fit between the empirical model and the actual data, whereas lower *R*^2^ values suggest the model is inadequate in explaining the relationship between variables. Our results showed that the *R*^2^ values for the response variables were all higher than 0.90, indicating that the regression models were suitable for describing the behavior of the system. The *R*^2^ values for the Hep2c, RD, and L2OB cell lines were found to be 0.9981, 0.9899, and 0.9973, respectively.

It is important to note that adding variables to the model will always increase *R*^2^, regardless of whether the additional variable is statistically significant or not. Therefore, a high *R*^2^ value alone does not necessarily indicate model adequacy. For this reason, it is more appropriate to consider an *R*^2^*adj* value above 90% when evaluating model adequacy [9]. The *R*^2^*adj* values for all responses were found to be higher than 0.98, indicating that insignificant terms were not included in the models.

Furthermore, the coefficient of variation (CV) describes the extent of data dispersion. Generally, CV values should not exceed 10% [16]. Our results showed that the CV values were less than 2% for all responses (Table 5), indicating high precision and reliability of the conducted experiments.

### 2.2. Kinetics of Extraction of BCs with Cytotoxic Activity

#### 2.2.1. Cytotoxic Activity Variation with Extraction Time

**Figure 1:**
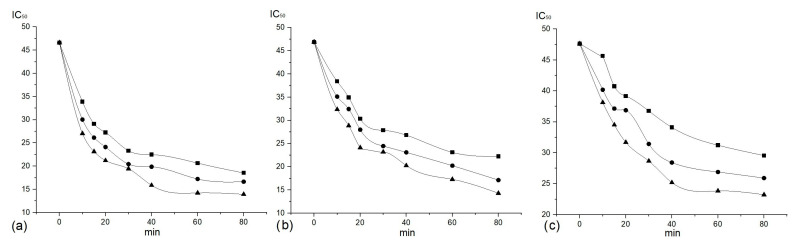
Variation in cytotoxic activity of extracts during the extraction process with (▲) 30%; (▪) 50%; and (•) 70% ethanol: (**a**) RD, (**b**) Hep2c, and (**c**) L2OB cell lines.

Figure 1 illustrates how cytotoxic activity changes during the extraction of bioactive compounds (BCs) from *Erica carnea* L. using different extraction solvents.

Analysis of the extraction curves for all the operative conditions selected in this study revealed two distinct stages of extraction. During the initial stage, which lasts approximately 20 min, the process is dominated by washing mechanisms. At this stage, the solvent rapidly solubilizes the bioactive compounds with cytotoxic activity that are readily available on the surface of the particles, resulting in a rapid increase in bioactive compound yield over time.

Following this initial phase, the extraction rate decreases substantially as diffusion becomes the predominant process.

#### 2.2.2. Kinetics of Extraction

Based on the observed mechanism, the experimental data on the kinetics of BC extraction (cytotoxic activity against Hep2c, RD, and L2OB cell lines as a function of time) were modeled using the unsteady-state diffusion model (Equation (6)) and the Ponomarev model (Equation (7)).

Both the unsteady-state diffusion model and the Ponomarev model, which account for the simultaneous washing and diffusion of BCs from plant material, describe the time-dependent variation in extractive substance yield throughout the entire extraction process. This approach represents an improvement in the modeling of BC extraction kinetics.

The kinetic model parameters were calculated from experimental data using the linear regression method applied to the appropriate linearized forms of the kinetic equations, as presented in Table 4.

The BCs washing coefficient (*b*) was found to be independent of extraction temperature. The model based on the Ponomarev equation predicted higher *b* values compared to the unsteady-state diffusion model, which predicted lower values of this coefficient (Table 4).

The *b* and *bʹ* values indicate that the extraction rate during the washing stage was highest with 30% ethanol, followed by 70% ethanol, while 50% ethanol exhibited the lowest washing rates for BCs with cytotoxic activity (Table 4). Similar differences were observed in the rates of the slow extraction phases (*k*, *kʹ*), which were significantly lower than those of the corresponding fast phases.

When comparing slow extraction rates, it was evident that different temperatures produced different reaction rates. An increase in temperature resulted in a higher slow extraction rate constant (*k*).

Table 4 shows the reaction rate of constants at different temperatures, where an increase in the reaction rate constant was observed with increasing temperature. According to Amarante et al. [17], higher extraction temperatures enhance solvent solubility and decrease the viscosity of both the solute and solvent, thereby facilitating the mass-transfer process.

#### 2.2.3. Comparison of the Kinetic Models

The criteria used to evaluate the models’ ability to describe the experimental data were the coefficients of determination (*R*^2^) and root mean square (RMS). A higher *R*^2^ value and a lower RMS value indicate a better fit of the model to the experimental data [18,19].

The statistical *R*^2^ and RMS results presented in Table 4 show that, regardless of the model applied, the individual *R*^2^ values were all higher than 0.8, while the RMS values were lower than ±10% for all extraction conditions.

Therefore, both evaluated models appear to be suitable for describing the extraction of BCs with cytotoxic activity against Hep2c, RD, and L2OB cell lines from *Erica carnea* L. using different ethanol concentrations. However, based on the *R*^2^ and RMS values, the unsteady-state diffusion model more accurately describes the extraction kinetics of bioactive compounds with cytotoxic activity than the Ponomarev model.

#### 2.2.4. Thermodynamic Study

Activation energy (*Ea*) represents the minimum amount of energy required for the extraction process to occur. Equation (6) is the linearized Arrhenius equation derived from Equation (5), which establishes the relationship between temperature (*T*) and the slow extraction rate (calculated based on the unsteady-state diffusion model).

Plotting ln k against 1/T produces a slope of −Ea/R and an intercept of ln A. The calculated activation energies for BC extraction were in the ranges of 10.52–26.57 kJ/mol for Hep2c, 6.97–11.95 kJ/mol for RD, and 4.92–17.45 kJ/mol for L2OB. These *Ea* values indicate that diffusion through a constrained boundary layer governs the rate of the process [20]. The obtained activation energy (*Ea*) values are relatively consistent with previously published research on the extraction of various oils and phenolic compounds [21,22].

The thermodynamic activation parameters, namely activation enthalpy (ΔH*), activation entropy (ΔS*), and activation Gibbs free energy (ΔG*), were calculated for each temperature for the extracted BCs using Equations (7) and (8).

Table 6 presents the average data for ΔH*, ΔS*, and ΔG* at temperatures of 30 °C, 40 °C, and 50 °C, with 30%, 50%, and 70% ethanol as the solvents. The values evaluated were as follows:

- Hep2c: ΔH* = 14.41 kJ/mol, ΔS* = −250.82 J/Kmol, ΔG* = 92.90 kJ/mol.
- RD: ΔH* = 6.77 kJ/mol, ΔS* = −273.81 J/Kmol, ΔG* = 92.77 kJ/mol.
- L2OB: ΔH* = 8.16 kJ/mol, ΔS* = −268.55 J/Kmol, ΔG* = 92.22 kJ/mol.

**Table 6:** Cytotoxic activity of *E. carnea* L. extracts.

Ethanol %	T (°C)	Hep2c Cells ^a^ IC_50_(μg/mL)	RD Cells ^b^ IC_50_(μg/mL)	L2OB Cells ^c^ IC_50_(μg/mL)
30	30	16.33 ± 0.55 ^d^	15.70 ± 0.89	25.21 ± 0.69
	40	15.23 ± 0.94	14.19 ± 0.71	24.26 ± 0.32
	50	14.29 ± 0.95	13.93 ± 0.65	22.23 ± 0.87
50	30	24.37 ± 0.12	22.44 ± 0.25	31.91 ± 0.43
	40	23.38 ± 0.49	21.40 ± 0.66	30.48 ± 0.21
	50	20.55 ± 0.23	17.49 ± 0.23	28.32 ± 0.74
70	30	22.22 ± 0.49	18.53 ± 0.44	29.55 ± 0.83
	40	21.36 ± 0.24	17.88 ± 0.87	28.87 ± 0.73
	50	20.24 ± 0.84	17.19 ± 0.92	27.88 ± 0.71
cis-DDP ^e^		0.94 ± 0.55	1.4 ± 0.97	0.72 ± 0.64

^a^ Cell line derived from human cervix carcinoma. ^b^ Cell line derived from human rhabdomyosarcoma. ^c^ Cell line derived from murine fibroblast. ^d^ Mean value ± 3SD. ^e^ Cis-diamminedichloroplatinum.

According to the obtained results, the positive values of activation enthalpy (ΔH*) indicate that the process is endothermic, meaning that an external source of energy is required to increase the energy contributing to the extraction yield to reach the state transition.

Regarding the entropy values, negative values were obtained under all experimental conditions. The negative ΔS* values suggest that the system becomes more ordered during extraction, likely due to complex formation. This implies that reacting species combined to form a state transition during the extraction process [23]. The strongly negative ΔS* values suggest a more complex transition and a lower extraction rate, as the internal energy level decreases during the transition.

The Gibbs free energy of activation (ΔG*) was used to assess the spontaneity of the extraction process at all tested temperatures. The positive ΔG* values indicate that the extraction processes are endergonic and non-spontaneous.

### 2.3. Biological Activity of Obtained Extracts

Previous studies have shown that the biological activity of plant extracts depends on the content of various phytochemicals, which are isolated through the application of suitable extraction techniques [5,24,25]. A significant number of anti-tumor compounds are primary and secondary plant metabolites, both in their natural and structurally modified forms. Plant extracts and their metabolites can interact with numerous target molecules within signaling pathways of malignantly transformed cells.

The results of this study confirm that extracts with strong antiproliferative potential can be obtained from plant material by using an appropriate solvent and extraction technique. The extracts obtained—prepared using different ethanol concentrations (30%, 50%, and 70%), at varying extraction temperatures (30 °C, 40 °C, and 50 °C), and with an extraction time of 80 min—were tested for cytotoxic, antioxidant, and antibacterial activities.

It is important to note that the same plant material (*Erica carnea* L.) used in this study was previously investigated by Veličković et al. [5], under different extraction conditions (96% ethanol, 22 °C, maceration over 7 days in a dry place). In that study, several phenolic acids were identified, with chlorogenic acid (0.132 mg/g) and vanillic acid (0.129 mg/g) being the most abundant, followed by ferulic acid (0.113 mg/g) and p-coumaric acid (0.108 mg/g). Other detected phenolic acids included p-hydroxybenzoic and rosmarinic acid. Among the flavonoids, the most prominent compounds were rutin (2.159 mg/g), quercetin (1.895 mg/g), luteolin (0.559 mg/g), and apigenin (0.321 mg/g).

In addition, that study reported a total phenolic content ranging from 98.86 to 144.12 mg GAE/g and a total flavonoid content from 19.18 to 26.24 mg RUE/g, depending on the extraction technique applied. These values confirm the high presence of bioactive polyphenols in the same plant matrix. Notably, the extract obtained using a maceration method similar to ours showed 118.14 mg GAE/g of total phenolics and 20.57 mg RUE/g of total flavonoids.

Even with different extraction conditions, the known compounds in *Erica carnea* likely play a key role in its strong cytotoxic, antioxidant, and antibacterial effects. Their previously established biological effects correspond well with the activities measured in our experiments, further reinforcing the potential of *Erica carnea* L. as a rich natural source of pharmacologically relevant compounds.

Moreover, in the study by Veličković et al. [5], the extracts obtained under those conditions showed higher IC_50_ values—with IC_50_ values of 32.28 µg/mL for Hep2c, 30.84 µg/mL for RD, and 28.51 µg/mL for L2OB—compared to the values reported in our work for the same cell lines. This difference may be attributed to the long maceration time and lack of temperature control, which could lead to degradation of sensitive phenolic compounds. Additionally, the use of 96% ethanol in their extraction protocol is likely less effective for phenolic compound recovery than the lower ethanol concentrations applied in our optimized method.

#### 2.3.1. Cytotoxic Activity

The cytotoxic activity of the obtained extracts against the three cell lines is presented in Table 6. The 30% ethanol extract exhibited the strongest activity against all three cell lines tested. Additionally, higher extraction temperatures corresponded to stronger cytotoxic activity of the extracts, regardless of ethanol concentration.

According to the US National Cancer Institute, the criterion for cytotoxic activity of a plant extract is IC_50_ < 30 µg/mL [26]. Based on this criterion, it can be concluded that most of the extracts meet the required standard.

The superior cytotoxic activity observed for extracts obtained at 30% ethanol and 50 °C likely reflects the enhanced extraction of moderately polar compounds, such as phenolic acids and flavonoids, which are known to induce apoptosis and inhibit tumor cell proliferation. These findings are consistent with previous reports highlighting the anticancer potential of these phytochemical classes.

Numerous studies have demonstrated the anticancer properties of various phenolic and polyphenolic compounds. For instance, quercetin and kaempferol have shown anticancer properties against various cell lines [27,28]. Moreover, hydroxycinnamic acids such as caffeic, ferulic, chlorogenic, and sinapic acids have exhibited inhibitory activity against the proliferation of cancer cell lines [28].

Previous studies have reported the presence of different phenolic acids and flavonoids in *E. carnea*, including rosmarinic acid, chlorogenic acid, p-coumaric acid, ferulic acid, caffeic acid, quercetin, rutin, kaempferol, and apigenin. The presence of these classes of compounds, as well as the individual compounds mentioned, likely explains the detected cytotoxic activity against the tested cell lines.

#### 2.3.2. Antibacterial Activity

The antibacterial activity of the prepared extracts was tested against eight different bacterial strains, and the results are presented in Table 7. Different strains exhibited varying sensitivity to the different extracts. Generally, the best results were observed for extracts prepared with 30% ethanol.

It is known that plants synthesize phenolic compounds in response to microbial infection, which may explain the antibacterial activity observed in the extracts [29,30]. Mori et al. [29] indicated that the presence of free hydroxyl groups attached to the phenyl ring of flavonoids is essential for antibacterial activity.

However, there is evidence suggesting that phenolic compounds are not the sole contributors to antibacterial activity. Other secondary metabolites, as well as potential synergistic effects, may also play a role and contribute to the observed antibacterial activity [31]. This may explain the high activity of *Erica carnea* L. extracts against most of the tested strains.

The antibacterial effects of the extracts are likely associated with the ability of phenolic compounds to disrupt bacterial cell membranes and interfere with enzymatic systems. The higher activity observed for lower ethanol concentration extracts supports the notion that extraction of polar phenolics is more efficient under these conditions, contributing to the observed antimicrobial potency.

#### 2.3.3. Antioxidant Activity

Antioxidant activity was assessed using five different assays: inhibition of lipid peroxidation, hydroxyl radical scavenging activity, DPPH and ABTS scavenging activities, and metal chelating activity. The results are presented in Table 4. The highest activity across all five assays was observed for extracts prepared with 30% ethanol, while the lowest activity was detected for extracts prepared with 50% ethanol.

The peroxidation of unsaturated fatty acids is a major cause of oxidative damage to cell membranes and other lipid-containing biological systems. All extracts demonstrated high activity against lipid peroxidation (Table 8). All extracts reduced hydroperoxide formation, with IC_50_ values between 19.44 and 27.60 µg/mL. Increasing the temperature from 30 to 50 °C resulted in extracts exhibiting significant differences in their ability to inhibit lipid peroxidation, with improvements ranging from 9.3% to 10.3%.

A similar trend was observed in the case of DPPH • radicals, where maximum antiradical activity was detected in the extract obtained at 50 °C. As the temperature increased, the extracts demonstrated higher activity towards DPPH radicals. The results obtained ranged from 16.55 to 25.03 µg/mL, indicating strong antiradical potential.

Hydroxyl radicals (OH •) are among the most reactive free radical species associated with tissue damage, degradation of proteins, insoluble lipids, carbohydrates, nucleic acids, and other essential biomolecules [32]. Due to their detrimental effects on living systems, the capacity of natural molecules to neutralize these radicals is of great importance.

All tested extracts demonstrated a high capacity to neutralize OH • radicals. The most potent extract in neutralizing OH • radicals was obtained at a temperature of 50 °C with 30% ethanol, while the lowest activity was measured for the sample obtained at 30 °C with 50% ethanol. The activity of all extracts was higher than the antioxidant activity of standard compounds, ascorbic and gallic acids.

The temperature of 50 °C also proved to be optimal in the case of ABTS • + radicals. Generally, the influence of extraction temperature on the scavenging ability toward long-living ABTS • + radical species was similar to the previous case. Extraction temperatures up to 50 °C produced extracts with increased activity, indicating that the extraction of antioxidant compounds was most efficient at this temperature. As in the previous case, the activity was comparable to that of BHT (Table 8).

The high antioxidant capacity of the extracts, particularly those prepared at 50 °C with 30% ethanol, may be attributed to the presence of phenolic compounds capable of donating electrons or hydrogen atoms to neutralize reactive oxygen species. The strong correlation between antioxidant and cytotoxic assays further suggests that these compounds play a dual role in cellular protection and antiproliferative activity.

Pearson’s correlation coefficients between antioxidant activity and cytotoxic activity of the obtained extracts from *Erica carnea* L. are shown in Table 9. The presented coefficients indicated particularly high correlations in most cases (*r* > 0.92), while all cases demonstrated strong correlations (*r* > 0.85).

These correlations suggest that the bioactive compounds present in the *Erica carnea* L. extracts are responsible for both antioxidant and cytotoxic activities. This conclusion is further supported by the correlation coefficients observed among the applied assays.

### 2.4. Mechanistic Considerations of Observed Bioactivities

Although the focus of this study was to evaluate the biological activities of *Erica carnea* extracts, it is useful to consider how these effects might occur. The strong cytotoxic activity observed could be related to compounds in the extract that affect cancer cell survival, possibly by triggering processes like oxidative stress or programmed cell death. The antioxidant effects are likely due to compounds that can easily donate electrons and help neutralize harmful molecules in cells. As for the antibacterial properties, certain components in the extracts may interfere with the structure or function of bacterial membranes, making it harder for the bacteria to grow or survive. While we did not explore these mechanisms in detail, the patterns in our results suggest that such actions are likely involved.

## 3. Materials and Methods

### 3.1. Chemicals and Reagents

All chemicals and reagents were of high analytical grade and obtained from Sigma (Sigma-Aldrich GmbH, Sternheim, Germany) and Sigma (St. Louis, MO, USA).

### 3.2. Plant Material

**Figure 2:**
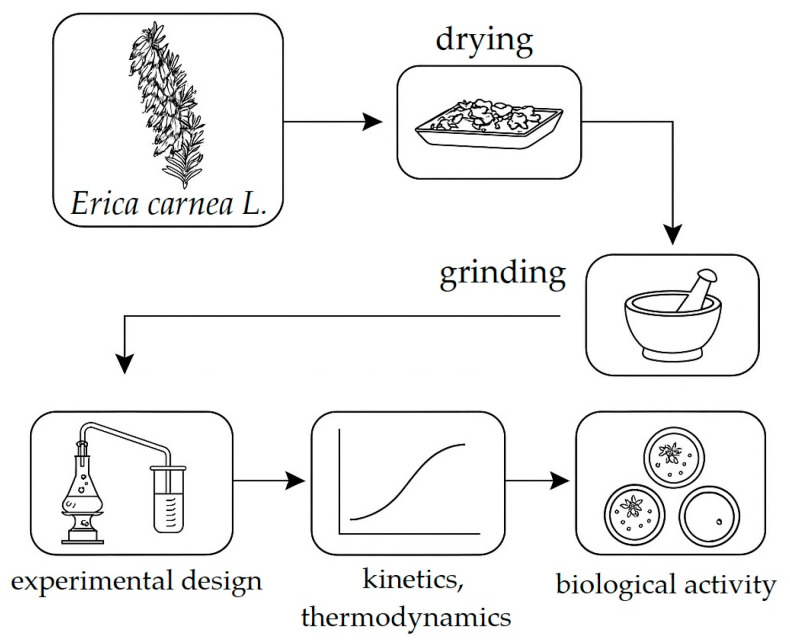
Preparation stages of *Erica carnea* extract for analysis.

Spring heath (*Erica carnea* L.) material was collected from the Čačak area (Republic of Serbia). Voucher specimens (*Erica carnea* L., Čačak area, determiner dr Milan Stanković, N° 126/017) are deposited at Institute of Biology, Faculty of Science, University of Kragujevac. The aerial parts of the plant were dried naturally in the shade with good air circulation for one month. The dried plant material was ground using a blender and stored in paper bags until further use. The extract preparation procedure is illustrated in Figure 2.

### 3.3. Preparation of the Extracts

Maceration was performed using the following procedure: Plant samples (2.5 g) were extracted using different ethanol concentrations (30%, 50%, 70%) as solvents. The extraction process was carried out under laboratory conditions at temperatures of 30 °C, 40 °C, and 50 °C for 80 min, with occasional shaking to enhance the maceration process.

After 80 min, the extract was filtered through filter paper (Whatman No.1, Whatman™, Cytiva, Maidstone, UK) and concentrated to dry mass using a rotary evaporator (Devarot, Elektromedicina, Ljubljana, Slovenia). The dried extracts were stored in dark glass bottles at 4 °C to prevent oxidative damage.

### 3.4. Extraction Procedure for Optimization Process

A 2^3^ full experimental design was employed to determine the optimal extraction conditions based on three independent variables: solvent concentration, extraction temperature, and extraction time. The independent variables were varied at two levels (−1, +1), resulting in eight experimental runs, with their actual values presented in Table 1 and Table 10.

The cytotoxic activity of the extracts against Hep2c, RD, and L2OB cell lines was defined as the response.

The experimental data were fitted by linear first-order polynomial Equation (4):

(4) $$y=b_{o}+b_{1}x_{1}+b_{2}x_{2}+b_{3}x_{3}+b_{12}x_{1}x_{2}+b_{13}x_{1}x_{3}+b_{23}x_{2}x_{3}+b_{123}x_{1}x_{2}x_{3}$$

where x_1_, x_2_, and x_3_ represent ethanol concentration, extraction time, and extraction temperature, respectively. The equation terms such as *x*_1_*x*_2_, *x*_1_*x*_3_, and *x*_2_*x*_3_ describe the interaction between two independent variables, while *x*_1_*x*_2_*x*_3_ represents the interaction of all three independent variables. *b*_0_ is the constant regression coefficient, *bᵢ* are the linear regression coefficients, and *bᵢⱼ* and *bᵢⱼₖ* are the regression coefficients of two-factor and three-factor interactions, respectively.

The regression coefficients were calculated using multiple linear regression. The statistical significance of the independent variables on the content of phenolic compounds and the quality of the model fit were evaluated at a confidence level of 95% (*p* < 0.05) using analysis of variance (ANOVA). If *p* < 0.05, the terms were considered statistically significant.

Statistical indicators such as the adjusted coefficient of determination (*R*^2^*adj*), coefficient of determination (*R*^2^), and coefficient of variation (CV) were used to assess model quality.

### 3.5. Kinetics of BCs Extraction

Plant samples (2.5 g) and the extraction solvent (aqueous ethanol solution at a concentration of 30% (*v*/*v*)) (50 mL) were placed in a series of Erlenmeyer flasks (250 mL) and left for 10, 15, 20, 30, 40, 60, and 80 min. The temperature was controlled and maintained at 30 ± 0.1 °C. After each time interval, the liquid extract was separated from the plant material by vacuum filtration.

The cytotoxic activity of the liquid extracts was determined. Each liquid extract was prepared in triplicate, and the results were averaged. The procedure was repeated using 50% and 70% ethanol at temperatures of 40 ± 0.1 °C and 50 ± 0.1 °C.

### 3.6. Determination of Biological Activity of Extracts

#### 3.6.1. Cytotoxic Activity

Cytotoxic activity was assessed according to a previously described method [33], using the well-established MTT (3-[4,5-dimethylthiazol-2-yl]-2,5-diphenyl tetrazolium bromide) assay [34]. The following cell lines were used: RD (human rhabdomyosarcoma cell line), Hep2c (human cervix carcinoma cell line—HeLa derivative), and L2OB (murine fibroblast cell line). The activity of the extracts obtained was measured against these cell lines.

Cells were cultured in Dulbecco’s Modified Eagle Medium (DMEM) supplemented with 10% fetal bovine serum (FBS) and 1% penicillin-streptomycin, and maintained at 37 °C in a humidified 5% CO_2_ atmosphere. Extracts were applied after 24 h of seeding, and the treatment lasted for 24 h. Although the doubling times were not experimentally determined in this study, literature reports suggest approximate doubling times of 22–30 h for these cell lines under standard conditions.

To ensure that ethanol used as a solvent had no cytotoxic effect, control samples with equivalent ethanol concentrations (≤1%) were included. No reduction in cell viability was observed in these controls.

IC_50_ values (μg/mL) represent the concentration needed to reduce cell survival by 50% compared to untreated controls [35]. The standard compound used was cis-diamminedichloroplatinum (cis-DDP). All experiments were performed in triplicate.

#### 3.6.2. Antioxidant Activity

The antioxidant activity of the obtained extracts was determined using several different, previously described assays, including lipid peroxidation assay [36], hydroxyl radical scavenging activity [37], DPPH radical scavenging activity [38], ABTS radical scavenging activity [39], and metal chelating activity [40]. Results for all assays were expressed as IC_50_ values in µg/mL.

#### 3.6.3. Antibacterial Activity

The antimicrobial potential of the investigated extracts was determined by measuring their antibacterial and antifungal activities according to previously described methods in the literature [41,42]. For the antibacterial tests, five different bacterial strains were used, while antifungal activity was assessed using two fungal species. Simultaneously, the activity of standard antimicrobial compounds Amracin (Sigma-Aldrich, St. Louis, MO, USA) and Nystatin (Merck KGaA, Darmstadt, Germany) was also measured.

Minimum inhibitory concentrations (MICs) of the extracts against the tested bacteria were determined using the microdilution method in 96-well microtiter plates. All tests were performed in Muller–Hinton broth (MHB). The extracts were dissolved, and seven different concentrations (19.53–625 μg/mL) were used to determine their antibacterial activity. For Amracin and Nystatin, the concentration range used for testing was 19.53–312.50 μg/mL.

As noted in Section 3.6.1, controls containing equivalent ethanol concentrations (≤1%) were included to exclude any solvent-related effects. No antimicrobial activity was observed in these control samples.

The plates were incubated for 24 h. Subsequently, color change was assessed visually. Any color change from purple to pink or colorless was recorded as positive. The lowest concentration at which a color change occurred was considered the MIC value. The average of three measurements was calculated, and the resulting value was reported as the MIC.

### 3.7. Modeling of BC Extraction Kinetics

The extraction kinetics was investigated using one physical model (unsteady-state diffusion model) and one empirical model (Ponomarev model). The kinetic parameters of the extraction are important for evaluating the bioactive compound extraction potential from *Erica carnea* L.

#### 3.7.1. Unsteady-State Diffusion Model

The unsteady-state diffusion model is expressed in Equation (5) and can be rearranged as linear Equation (3) [43,44]:

(5) $${CA}_{t}/{CA}_{o}=\left(1-b\right)e^{-kt}$$

(6) $$ln\left({CA}_{t}/{CA}_{o}\right)=ln\left(1-b\right)-kt$$

where ${CA}_{t}$ is the cytotoxic activity against three cell lines (Hep2c, RD, L2OB) in the liquid extract during the extraction (IC_50_ μg/mL), *CA_o_* is the initially cytotoxic activity, *k* is the slow extraction coefficient of the unsteady-state diffusion model (1/min), and *b* is the washing coefficient of the unsteady-state diffusion model.

#### 3.7.2. Ponomarev Model

The Ponomarev model, being very simple, is frequently used to model the slow extraction period [28]:

(7) $$\frac{{CA}_{o}-{CA}_{t}}{{CA}_{o}}=b^{\prime }+k^{\prime }t$$

where *k^ʹ^* is the slow extraction coefficient of the Ponomarev model and *b^ʹ^* is the washing coefficient of the Ponomarev model.

Kinetic parameters were calculated from linearized forms of the models (Equations (6) and (7)) using linear regression. The best fit among the kinetic models was evaluated using coefficient of determination (R^2^) and root mean square (RMS) [45]. The higher the value of R^2^ and the lower the values of the RMS, the better will be the goodness of fit [45].

### 3.8. Determination of Thermodynamic Parameters

The Arrhenius equation was used for the calculation of the activation energy, which was used to describe the relationship between *k*, the slow extraction coefficient of the unsteady-state diffusion model, and the temperature. It is expressed in Equation (8), and can be rearranged as linear Equation (9) [46]:

(8) $$k=Ae^{-E_{a}/RT}$$

(9) $$lnk=\frac{-E_{a}}{R}\left(\frac{1}{T}\right)+lnA$$

where *E_a_* is the activation energy (kJ/mol), *R* is the gas constant, and *T* is the absolute temperature (K).

A plot of lnk vs. 1/T gives a straight line whose slope represents the activation energy of extraction –Ea/R, and whose intercept is the Arrhenius constant, ln A. The activation thermodynamic parameters were calculated according to the equations:

(10) $$∆H^{*}=E_{a}-RT$$

(11) $$∆G^{*}=∆H^{*}-T∆S^{*}$$

where ΔH* is the activation enthalpy, ΔS* is the activation entropy, and ΔG* is the activation free energy or Gibb’s energy.

## 4. Conclusions

This study highlights *Erica carnea* L. as a promising natural source of multifunctional bioactive compounds. The tested extracts demonstrated strong antioxidant, cytotoxic, and antibacterial activity, suggesting their potential for use in health-related applications. By optimizing extraction conditions and applying kinetic and thermodynamic modeling, we gained valuable insight into the behavior of these compounds during extraction. Future work should focus on identifying the individual active components and further investigating their mechanisms of action and therapeutic relevance.

## Figures and Tables

**Table 1:** Experimental design matrix with experimental data.

No	Independent Variables			Measured Responses					
	x_1_ (%)	x_2_ (°C)	x_3_ (min)	Hep2c-Y_ex_^a^	Hep2c-Y_pred_	RD-Y_ex_	RD-Y_pred_	L2OB-Y_ex_	L2OB-Y_pred_
1	30 (−1)	30 (−1)	20 (−1)	0.454	0.452	0.558	0.552	0.328	0.318
2	70 (+1)	30 (−1)	20 (−1)	0.442	0.438	0.421	0.418	0.181	0.167
3	30 (−1)	50 (+1)	20 (−1)	0.465	0.467	0.57	0.576	0.328	0.348
4	70 (+1)	50 (+1)	20 (−1)	0.450	0.439	0.439	0.442	0.192	0.181
5	30 (−1)	30 (−1)	80 (+1)	0.669	0.666	0.692	0.692	0.586	0.575
6	70 (+1)	30 (−1)	80 (+1)	0.606	0.609	0.602	0.612	0.391	0.411
7	30 (−1)	50 (+1)	80 (+1)	0.683	0.685	0.716	0.716	0.603	0.589
8	70 (+1)	50 (+1)	80 (+1)	0.659	0.656	0.647	0.637	0.448	0.452

^a^ Y-yield of cytotoxic activity.

**Table 2:** Regression coefficients of the first-order polynomial.

			Response			
Coefficient	Hep2c	PC (%) ^a^	RD	PC (%)	L2OB	PC (%)
b_0_	0.5535	-	0.5806		0.3792	
b_1_	−0.0142	9.54	−0.0534	30.83	−0.0762	31.28
b_2_	0.0087	5.85	0.0124	7.16	0.0135	5.54
b_3_	0.1007	67.67	0.0836	48.27	0.1277	52.42
b_12_	0.0025	1.68	0.0034	1.96	0.0035	1.44
b_13_	−0.0075	5.04	0.0136	7.85	0.0112	4.60
b_23_	0.0080	5.37	0.0049	2.83	0.0050	2.05
b_123_	0.0072	4.85	0.0019	1.10	0.0065	2.67

^a^ Percentage Contribution.

**Table 3:** Results of the analysis variance.

Cell Lines	SOV	x_1_	x_2_	x_3_	x_1_x_2_	x_1_x_3_	x_2_x_3_	x_1_x_2_x_3_	Error	Total
Hep2c	SS ^1^	0.00162	0.00061	0.08120	0.00005	0.00045	0.00051	0.00042	0.00016	0.08502
	Df ^2^	1	1	1	1	1	1	1	8	15
	MS ^3^	0.00162	0.00061	0.08120	0.00005	0.00045	0.00051	0.00042	0.00002	0.00567
	F-Value	81.00	30.50	4060.0	2.50	22.50	25.50	21.00		
	*p*-Value	0.000190	0.000559	<0.00001	0.152502	0.001458	0.000990	0.001796		
	R^2^ = 0.9981; R_adj_^2^ = 0.9964; CV (%) = 0.81									
RD	SS	0.02279	0.00122	0.00559	0.00009	0.00148	0.00019	0.00003	0.00032	0.03171
	Df	1	1	1	1	1	1	1	8	15
	MS	0.02279	0.00122	0.00559	0.00009	0.00148	0.00019	0.00003	0.00004	0.00211
	F-Value	569.75	30.50	1398.6	2.25	37.00	4.75	0.75		
	*p*-Value	<0.00001	0.000559	<0.00001	0.172003	0.000295	0.060915	0.411694		
	R^2^ = 0.9899; R_adj_^2^ = 0.9810; CV (%) = 1.09									
L2OB	SS	0.04651	0.00146	0.13056	0.00010	0.00101	0.00020	0.00034	0.00048	0.18066
	Df	1	1	1	1	1	1	1	8	15
	MS	0.04651	0.00146	0.13056	0.00010	0.00101	0.00020	0.00034	0.00006	0.01204
	F-Value	775.16	24.33	2176.0	1.6666	16.833	3.333	5.666		
	*p*-Value	<0.00001	0.001146	<0.00001	0.232845	0.003424	0.105337	0.044522		
	R^2^ = 0.9973; R_adj_^2^ = 0.9950; CV (%) = 1.98									

SOV-Source of variation; ^1^ Sum of squares; ^2^ Degree of freedom; ^3^ Mean of square.

**Table 4:** Unsteady-state diffusion model and Ponomarev model parameters for extraction of bioactive compounds with cytotoxic activity against Hep2c, RD, and L2OB cell lines.

C. Lines/ Ethanol (%)	T (°C)	Unsteady b	-Stare k	Diffusion RMS	Model R^2^	Ponomarev bʹ	Model kʹ	RMS	R^2^
Hep2c	30 °C	0.441	2.46 × 10^−3^	5.52	0.9465	0.450	2.35 × 10^−3^	7.38	0.9048
30	40 °C	0.439	2.64 × 10^−3^	4.66	0.9569	0.457	2.45 × 10^−3^	6.62	0.9189
	50 °C	0.425	2.87 × 10^−3^	4.38	0.9661	0.442	2.61 × 10^−3^	6.84	0.9278
50	30 °C	0.352	1.33 × 10^−3^	1.27	0.8598	0.358	1.66 × 10^−3^	4.98	0.826
	40 °C	0.345	1.60 × 10^−3^	1.12	0.8987	0.353	1.94 × 10^−3^	4.97	0.866
	50 °C	0.345	1.87 × 10^−3^	1.01	0.915	0.359	2.15 × 10^−3^	4.94	0.883
70	30 °C	0.414	1.76 × 10^−3^	5.14	0.9019	0.436	1.78 × 10^−3^	5.46	0.8645
	40 °C	0.417	1.90 × 10^−3^	3.48	0.9508	0.438	1.87 × 10^−3^	4.61	0.9222
	50 °C	0.405	2.28 × 10^−3^	6.8	0.8972	0.433	2.20 × 10^−3^	8.31	0.8363
RD 30	30 °C	0.613	2.08 × 10^−3^	5.75	0.8606	0.621	1.69 × 10^−3^	7.48	0.8219
	40 °C	0.613	2.24 × 10^−3^	6.78	0.849	0.62	1.74 × 10^−3^	8.32	0.8014
	50 °C	0.542	2.79 × 10^−3^	6.65	0.8949	0.561	1.88 × 10^−3^	7.71	0.8793
50	30 °C	0.374	1.42 × 10^−3^	3.34	0.9489	0.378	1.40 × 10^−3^	3.53	0.9322
	40 °C	0.391	1.58 × 10^−3^	0.99	0.8887	0.402	1.53 × 10^−3^	4.45	0.8753
	50 °C	0.415	1.78 × 10^−3^	1.15	0.8769	0.424	1.68 × 10^−3^	5.79	0.84
70	30 °C	0.386	1.91 × 10^−3^	3.76	0.9536	0.406	1.62 × 10^−3^	5.32	0.9193
	40 °C	0.389	2.08 × 10^−3^	5.63	0.9212	0.409	1.71 × 10^−3^	7.35	0.8772
	50 °C	0.401	2.18 × 10^−3^	4.95	0.9442	0.424	1.78 × 10^−3^	7.09	0.8979
L2OB 30	30 °C	0.255	3.27 × 10^−3^	2.91	0.9781	0.306	3.41 × 10^−3^	4.89	0.9543
	40 °C	0.229	3.48 × 10^−3^	2.68	0.9888	0.287	3.65 × 10^−3^	5.01	0.9635
	50 °C	0.202	3.69 × 10^−3^	3.09	0.9942	0.267	3.89 × 10^−3^	5.6	0.9826
50	30 °C	0.089	2.11 × 10^−3^	2.28	0.9803	0.12	2.75 × 10^−3^	2.09	0.9803
	40 °C	0.08	2.45 × 10^−3^	3.17	0.9847	0.121	3.21 × 10^−3^	1.98	0.9866
	50 °C	0.092	2.69 × 10^−3^	3.79	0.9769	0.143	3.61 × 10^−3^	3.69	0.9882
70	30 °C	0.154	1.74 × 10^−3^	1.92	0.9742	0.172	2.62 × 10^−3^	2.79	0.9551
	40 °C	0.139	2.17 × 10^−3^	2.24	0.9799	0.166	3.11 × 10^−3^	3.48	0.9571
	50 °C	0.156	2.67 × 10^−3^	3.34	0.9669	0.156	3.64 × 10^−3^	4.64	0.9421

**Table 5:** The activation thermodynamic parameters for extraction of compounds with cytotoxic activity against three cell lines with different ethanol concentration at different temperatures.

Cell Lines	Ethanol %	T °C	E_a_ kJmol^−1^	ΔH* kJmol^−1^	ΔS* JK^−1^mol^−1^	ΔG* kJmol^−1^
Hep2c	30	30		8.01	−271.28	90.20
		40	10.52	7.92	−272.02	93.07
		50		7.84	−271.81	95.63
	50	30		11.37	−262.50	90.91
		40	13.89	11.29	−262.70	93.51
		50		11.20	−263.04	96.17
	70	30		24.25	−215.54	89.36
		40	26.57	23.97	−218.29	92.21
		50		23.88	−220.22	95.02
RD	30	30		9.41	−256.24	89.78
		40	11.93	9.33	−266.15	92.63
		50		9.25	−265.77	95.09
	50	30		6.69	−277.41	90.74
		40	9.21	6.60	−277.76	93.54
		50		6.52	−277.95	96.30
	70	30		4.45	−280.26	89.26
		40	6.97	4.37	−281.25	92.40
		50		4.29	−281.49	95.21
L2OB	30	30		2.40	−284.50	88.61
		40	4.92	2.32	−284.88	91.49
		50		2.24	−285.15	94.34
	50	30		7.39	−271.90	89.77
		40	9.91	7.31	−271.87	92.40
		50		7.22	−272.30	95.18
	70	30		14.93	−248.50	90.23
		40	17.45	14.85	−248.78	92.72
		50		14.77	−249.05	95.21

**Table 7:** Antimicrobial activities (μg/mL) of *Erica carnea* L. extracts.

Stain	MIC	μg/mL									
	30% Ethanol			50% Ethanol			70% Ethanol			A ^a^	N ^b^
	30 °C	40 °C	50 °C	30 °C	40 °C	50 °C	30 °C	40 °C	50 °C		
*Staphylococcus aureus* ATCC 25923	19.53	19.53	19.53	156.25	156.125	78.125	39.1	39.1	39.1	39.1	
*Klebsiella pneumoniae* ATCC 13883	19.53	19.53	19.53	156.25	156.25	78.125	78.125	39.1	39.1	19.53	
*Escherichia coli* ATCC 25922	19.53	19.53	19.53	39.1	39.1	19.53	19.53	19.53	19.53	39.1	
*Proteus vulgaris* ATCC 13315	19.53	19.53	19.53	78.125	78.125	78.125	39.1	39.1	39.1	19.53	
*Proteus mirabilis* ATCC 14153	19.53	19.53	19.53	39.1	19.53	19.53	19.53	19.53	19.53	39.1	
*Bacillus subtilis* ATCC 6633	19.53	19.53	19.53	312.5	312.5	78.125	39.1	19.53	19.53	39.1	
*Candida albicans* ATCC 10231	19.53	19.53	19.53	156.25	156.25	78.125	78.125	78.125	78.125		19.53
*Aspergillus niger* ATCC 16404	19.53	19.53	19.53	39.1	19.53	19.53	19.53	19.53	19.53		39.1

^a^ A—Amracin; ^b^ N—Nystatin.

**Table 8:** Antioxidant activity of extracts from *Erica carnea* L. at different ethanol concentrations and temperatures.

Ethanol %	T °C	LP ^1^ IC_50_ (μg/mL)	DPPH ^2^ IC_50_ (μg/mL)	MC ^3^ IC_50_ (μg/mL)	OH ^4^ IC_50_ (μg/mL)	ABTS ^5^ IC_50_ (μg/mL)
	30	21.44 ± 0.55	19.35 ± 0.45	8.76 ± 0.84	13.54 ± 0.63	13.08 ± 0.13
30	40	20.01 ± 0.96	18.45 ± 0.23	7.66 ± 0.39	13.21 ± 0.61	12.61 ± 0.07
	50	19.44 ± 0.27	16.55 ± 0.50	6.98 ± 0.89	13.09 ± 0.55	12.17 ± 0.67
	30	27.60 ± 0.12	25.03 ± 0.10	16.01 ± 0.16	19.42 ± 0.11	17.41 ± 0.12
50	40	26.42 ± 0.60	24.43 ± 0.52	13.48 ± 0.14	17.19 ± 0.72	17.13 ± 0.92
	50	25.25 ± 0.62	23.98 ± 0.17	12.45 ± 0.15	16.92 ± 0.10	16.83 ± 0.34
	30	24.77 ± 0.21	22.14 ± 0.33	11.45 ± 0.13	14.22 ± 0.80	16.06 ± 0.21
70	40	24.23 ± 0.66	21.09 ± 0.98	10.98 ± 0.38	14.02 ± 0.81	15.51 ± 0.87
	50	22.54 ± 0.99	20.94 ± 0.99	10.52 ± 0.08	13.97 ± 0.20	14.21 ± 0.82
Gallic acid		255.43 ± 11.68	36.34 ± 0.20		59.14 ± 1.10	7.34 ± 0.21
Ascorbic acid		>1000			160.55 ± 2.31	2.39 ± 0.93
BHT		1.00 ± 0.23			33.92 ± 0.79	19.32 ± 0.72
α-tocopherol		0.48 ± 0.05				

^1^ lipid peroxidation assay; ^2^ DPPH radical scavenging activity; ^3^ Metal chelating activity; ^4^ Hydroxyl radical scavenging activity; ^5^ ABTS scavenging activity.

**Table 9:** Pearson’s correlation coefficients among antioxidant activity and cytotoxic activity.

Test	LP.	DPPH	MC	OH	ABTS	Hep2c	RD	L2OB
LP	1							
DPPH	0.9793	1						
MC	0.9808	0.9658	1					
OH	0.9425	0.8867	0.9286	1				
ABTS	0.9393	0.9716	0.9518	0.8508	1			
Hep2c	0.9579	0.9670	0.9587	0.8713	0.9930	1		
RD	0.9832	0.9491	0.9802	0.9325	0.9337	0.9506	1	
L2OB	0.9774	0.9849	0.9868	0.8871	0.9857	0.9867	0.9662	1

**Table 10:** Parameters and levels used in the 2^3^ factorial design study.

Parameters	Level (−)	Level (+)
Solvent concentration, % (x_1_)	30	70
Extraction temperature, °C (x_2_)	30	50
Extraction time, min (x_3_)	20	80
Extraction method	Maceration	

## Data Availability

The original contributions presented in the study are included in the article; further inquiries can be directed to the corresponding author.
